# Molecular and Vegetative Compatibility Groups Characterization of *Aspergillus flavus* Isolates from Kenya

**DOI:** 10.3934/microbiol.2020015

**Published:** 2020-07-31

**Authors:** Alfred Mitema, Naser Aliye Feto

**Affiliations:** 1 OMICS Research Group, Department of Biotechnology, Vaal University of Technology, Vanderbijlpark 1911, South Africa; 2 School of Biological Sciences, University of Nairobi, Nairobi, Kenya

**Keywords:** *Aspergillus flavus*, aflatoxicosis, morphotype, vegetative compatibility group, heterokaryon

## Abstract

The genus *Aspergillus* contains diverse species and the identification is complicated. Vegetative compatibility groups (VCGs) and molecular mechanisms were deployed to study the species. The study was randomly conducted in four counties in Kenya based on the history of aflatoxicosis and maize cultivation. Thirty-seven *Aspergillus flavus* isolates from Nandi, Kisumu, Homa Bay and Makueni were characterized to determine their taxonomic status based on their VCGs and genotypes. A phylogenetic analysis of ITS1 and ITS2 sequences of the isolates investigated revealed ITS primers discriminating some of the *A. flavus* isolates as 100% sequence identity to the *RefSeq*. *Nit* mutants' complementation test revealed strong heterokaryon incompatibility between isolates of Nandi region (67%) and Makueni (33%). The trend based on VCGs and molecular findings showed high incidence of toxigenic *A. flavus* in Makueni, which could be the reason why the region frequently experiences chronic aflatoxicosis incidences over the last few decades as compared to other regions. Interestingly, we have discovered all S and L-morphotypes including the rare S/L-morphotypes, which implies that Kenya is home to all morphotypes of *A. flavus*. Thus, the analysis provides a deeper understanding of the taxonomic relationship between the *A. flavus* isolates and could help contextualise the data obtained for each isolate with respect to VCG genetic diversity and genotypes. Determining the primary causal agents of aflatoxin contamination is critical for predicting risk of contamination events and designing and implementing effective management strategies.

## 1. Introduction

Aflatoxin poisoning has been reported in many parts of the world in domestic and non-domestic animals, and other non-human primates [1]. Aflatoxin poisoning has led to serious economic losses through loss of livestock and poultry following consumption of contaminated feed or loss of income from local or international markets [2]. *Aspergillus* species (*Aspergillus flavus* and *Aspergillus parasiticus*) have been described on morphological and phenotypic parameters such as colony colour, diameter and size, the texture of conidia, the structure of conidiophores and sclerotia size.

Aflatoxin-production ability usually are preserved within a Vegetative Compatibility Group [3]–[5]. Vegetative compatibility groups (VCG) is a genetically determined ability in which isolates of the same fungal species anastomose and form stable heterokaryons, during which genetic material may transfer from one isolate to another [6].

*Aspergillus flavus* populations designated as the L and S-morphotypes based on colony and sclerotia morphology characteristics can be further subdivided into VCGs by a heterokaryon (vegetative) incompatibility system [6]. Heterokaryon incompatibility is a genetic phenomenon (*het* or *vic* loci) that limits heterokaryosis amongst entities which differ at one or more *het* or *vic* loci [7]. Heterokaryon incompatibility is circumvented only during sexual recombination [8]. Successful mating between parents of opposite mating type may occur even if they belong to different chemotypes or VCG. The determination of the VCG of a specific *A. flavus* strain is done by complementation tests with nitrate non-utilizing auxotroph's [9]–[13]. Members of the same VCG are presumed to have the same clonal lineage [9]–[11],[13], and the similarity of aflatoxin production is greater within a given VCG than between VCGs.

VCG analysis also has been used to evaluate genetic diversity within *A*. *flavus* populations and multiple VCGs can be recovered from the same geographic region including fields or crops from which isolates were obtained [4],[8],[11],[14]–[16].

Asao [17], observed that species classification in *Aspergillus* section *Flavi* is challenging due to extensive divergence and genetic variability. In contrast, molecular approaches for differentiating species in *Aspergillus* that rely on DNA sequence based techniques result in more robust species identifications [12],[18].

The ITS regions have been used in both ecological and molecular systematic investigations of fungi which has led to a repository of more than 200,000 Sanger-derived fungal ITS sequences in nucleotide databases such as GenBank [19]. The ITS regions of fungi vary, roughly, between 450–750 bp in length and consist of 3 sub-regions: an intercalary 5.8S gene (highly conserved) and more variable ITS1 and ITS2 spacer regions. The spacer regions provide resolution within the genus, often to the species level [20]. These genomic regions are being used as the official DNA barcode for classification in fungal studies [21].

Frisvad *et al.* [22] and Pildain *et al.* [23] both demonstrated that multiple species in *Aspergillus* section *Flavi* cannot be completely resolved based solely on morphological characters. Moreover, the ability to produce aflatoxin is not a useful character in discriminating species within *Aspergillus* section *Flavi*. Besides, Varga *et al.* [5] reported that many strains can lose their ability to produce aflatoxins overtime. The study sites were selected as Nandi, Kisumu, Homa Bay and Makueni counties of Kenya based on the climatic condition that is conducive for aflatoxin production and history of consistent aflatoxin incidence in the area [24],[25]. Therefore, the objective of the current study was to evaluate VCG genetic diversity and variation among the isolates and to conduct phylogenetic analysis of the *A. flavus* strains.

## 2. Materials and methods

### 2.1. Administrative study sites

**Figure 1.**
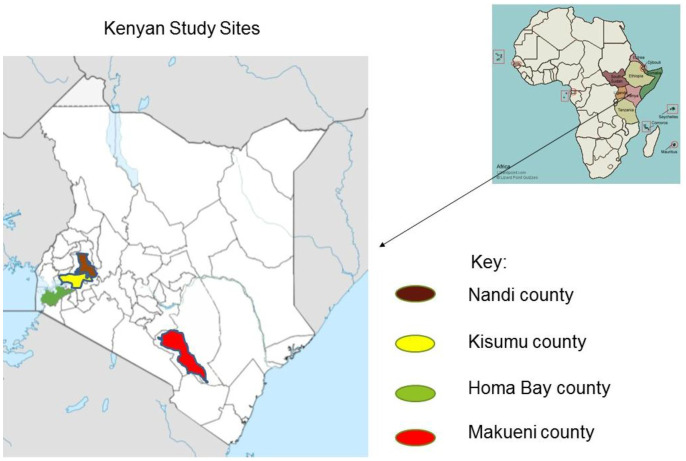
Map of Kenya showing four administrative counties in different climatic regions sampled out of 47; Makueni, Nandi, Homa Bay and Kisumu.

Among the four administrative counties (Figure 1) where sampling was conducted, Makueni is located in a drought-prone semi-arid zone of the former eastern province of Kenya at an elevation of between 800–1700 m above sea level. Additionally, Makueni has an annual rainfall between 300–600 mm and mean temperature of 24 °C [24],[25]. The county has two maize planting seasons, from March to May and from October to December where the weather pattern is characterized by extreme wet and hot conditions [24].

Nandi county is located in the former Rift Valley province of Kenya. Rainfall months extend from March to June, in which lengthy, heavy rains occur while the short rainfall months are from September to November [24]. The average temperature is 20 °C, with the highest temperature recorded in December and January 23 °C and the lowest, 12 °C, occurred in July and August.

Homa Bay and Kisumu counties are in the former Nyanza province of Kenya. Homa Bay county has an average temperature range of 21–35 °C. Kisumu county has an annual relief rainfall of between 1200–1300 mm and a mean annual temperature 23–35 °C. Nandi, Kisumu and Homa Bay counties have only one planting season from February to April.

### 2.2. Sampling

Samples were collected randomly in the months of November and December 2013. The identified sites were based on previous history of cultivation of maize. We used diagonal transect method and applied it to sample households as previously described [26],[27]. We compensated the participants identified based on the current market rates. Approximately two-three hundred grams of shelled corn kernels, or 2–3 cobs, were collected from each individual household thereafter stored in sterile brown paper bags and sealed in sterile zipped plastic polythene bags. The collected corn kernels were stored at 4 °C in a coolant container before transport to the Mycology Laboratory at the University of Nairobi, Kenya for further analysis.

### 2.3. Chemicals and reagents

Potato dextrose agar (PDA), yeast extract, sodium chloride, ammonium acetate and tryptone were from Merck chemicals (USA). Mycological peptone, malt extract agar (MEA), agar, water agar, ethanol, sucrose, Whatman No. 1 filter paper and sodium hypochlorite were from Sigma-Aldrich (USA). *Not*1 enzyme, *Taq* DNA Polymerase and detection kits were from Thermo™ Scientific (USA). Ampicillin, Isoprophylthio-β-D-galactoside (IPTG), chloroform, agarose gel, 5-bromo-chloro-3-indolyl-β-D-galactopyranoside (X-gal), isoamyl alcohol, glass beads and ethylene-diamine-tetra acetic acid (EDTA) were from Sigma-Aldrich (USA). Pure and ultra-pure water was from Molecular and Cell Biology laboratory, UCT (Millipore LTD, Bedford, MA, USA). Ligations were performed with the pGEM^®^ T Easy vector and T4 DNA Ligase kit (Promega Corporation, USA).

### 2.4. Fungal cultivation and isolation of pure colonies of *A. flavus*

The techniques followed were based on previous methods of Mitema and Okoth *et al.* [13],[28]. Briefly, the kernels were plated in triplicates on quarter strength PDA (Sigma Aldrich, USA) and incubated at 30 °C for 3 days. Kernels observed with fungal growth, in different shades of green, yellowish brown, black or cottony white, were transferred onto full strength PDA plates to obtain pure colonies. The pure colonies were single-spored, cultured in Water Agar (WA) (Sigma Aldrich, USA) and incubated at 25 °C for 24 h. Single growing mycelia were sub-cultured onto fresh PDA plates for further molecular analysis.

### 2.5. Testing of Vegetative Compatibility groups

To determine the diversity and distribution of VCGs across different agro-ecological zones, 37 isolates of *A. flavus* were used for VCG tester-pair development. The tester pair was obtained from Fungal Reference Library, Mycology Laboratory, University of Nairobi, Kenya. Nitrate non-utilizing (*nit−*) mutants were generated using a modified method of Bayman and Cotty [30]. Briefly, fungal isolates were grown on a selective medium [Czapek–Dox broth (Difco)] containing 25 g l^−1^ potassium chlorate, 50 mg l^−1^ rose bengal and 20 g l^−1^ agar with pH adjusted to 7. The selective medium was inoculated with a conidial suspension of *A. flavus* in a well at the centre of a 9 cm Petri plate. Cultures were incubated at 30 °C, and margins of colonies with restricted growth were periodically examined for fast growing sectors containing sparse mycelium. Hyphal tips from sectors arising from different colonies were transferred to Petri plates containing Czapek–Dox broth with 15 g l^−1^ potassium chlorate and 20 g l^−1^ agar with pH adjusted to 7 to stabilize the mutants. The *nit−*mutant phenotypes, *niaD−*(defective in the structural gene for nitrate reductase), *nirA−* (defective in the nitrate reductase) and *cnx−* (defective in the molybdenum cofactor) were determined by growing the mutants on a medium with nitrite, hypoxanthine or ammonium as sources of nitrogen as previously described [30],[31]. A complementary pair of nitrate non-utilizing auxotrophs composed of either a *niaD−* and a *cnx−* or a *niaD−* and a *nirA−* mutant was obtained for each isolate, and complementary pairings were first conducted to establish self-compatibility [30]. Complementary pairs of mutants from an isolate were used as tester pairs and complementation with one or both of the tester mutants of a VCG-defined membership in that VCG [11].

The generated *nit-* mutants from the isolates were used to determine the distribution of VCGs in the areas studied. Subsequently, the *nit−* mutants were used to perform complementation test with each VCG tester-pair. Complementation tests were conducted by placing 10 µL of a spore suspension of each member of a VCG-defining tester-pair and a *nit−* mutant (unknown phenotype) of one of the isolates in 3 mm wells cut into complementation medium. The wells were arranged approximately 15 mm apart in a triangular pattern so that each tester may react with both the *nit−* mutant and the other tester. The Petri plates were incubated at 30 °C from 7 to 14 days. Compatibility was identified by a line of prototrophic growth, where the mycelia interacted. This interaction was frequently associated with formation of sclerotia. Inter-location complementation was determined following the methods Atehnkeng *et al.* [11].

### 2.6. ITS1 and ITS2 Primer design

Oligonucleotide primer pairs (F-5′-TCA TTA CCG AGT GTT GGG TTC CTA G-3′ and R-5′-GGT CAA CCT GGA AAA GAC TGA TTT G-3′) were designed in Primer3 ver. 4.0 programme [32] based on sequence alignment of the ITS1 and ITS2 regions of *A. flavus* strains retrieved from NCBI (http://www.ncbi.nlm.nih.gov/). The secondary structure formation for the primers were evaluated in DNAMAN software ver. 6.0 (Lynnon LLC., USA) and further verified in OligoAnalyzer Tool version 3.1 (Integrated DNA Technologies, Inc., USA). The standard methods were used to synthesize the primers as provided by the Synthetic DNA Laboratory (MCB, University of Cape Town in South Africa). The PCR analysis was performed (GeneAmp PCR system 9700, Applied Biosystems) to detect both non-specific and specific amplification.

### 2.7. Genomic DNA isolation

*Aspergillus flavus* mycelia from 7-day old cultures were scrapped off the plates, flash frozen and ground in liquid nitrogen in a sterile mortar with a pestle prior to DNA extraction. Fungal DNA was recovered from ground cultures (100 mg) by using a ZR Fungal/Bacterial DNA Kit (Zymo Research, USA) according to manufactureŕs instructions. The DNA yield and integrity were evaluated, using a NanoDrop ND-1000 spectrophotometer (NanoDrop Technologies, USA) and further assessed on 1% (w/v) agarose/EtBr gel run at 80 volts for 45 min. The DNA was diluted to 50 ng/µL, tested for suitability for PCR amplification and, stored for further analysis at –80 °C.

### 2.8. Amplicon assessments of the fungal gDNA

PCR amplification was performed in a 20 µL reaction mixture containing 10 × 25 (1.5) mM MgCl_2_ buffer, 10 mM dNTPs, 5 U/µL Kapa Taq DNA polymerase (Kapa Biosystems Ltd., UK), 1 µL of template DNA and 10 µM of ITS1 and ITS2 forward and reverse primers [12]. The amplification procedure comprised of a pre-denaturation step at 96 °C for 3 min, followed by 30 cycles of denaturation at 96 °C for 30 s, annealing at 55 °C for 1 min and extension at 72 °C for 1 min, plus a final at 72 °C elongation for 5 min before being stored at 4 °C for 1 min. The integrity of PCR products was assessed on 1% (w/v) agarose/EtBr gel run at 80 volts for 45 min. One kilo base pair (1 kb) DNA ladder (Promega BioSciences, CA, USA) was used to detect the product sizes visualised under low radiation ultraviolet (UV) trans-illuminator (ChemiDoc™ XRS+ Bio-Rad ver.5.1, USA).

### 2.9. Gene cloning, plasmid DNA extraction and quantification

Purified PCR products were cloned using a 1:3 vector/insert ratio into the pGEM®-T-Easy vector (Promega Corporation, USA) according to the manufacturer's instructions. Ligated plasmid DNA was transformed into chemically competent *E. coli DH5α* cells (E. cloni ™, Lucigen, WI). Transformed cells were selected on LB agar plates (based on blue/white screening) with 100 µg/mL ampicillin, 80 µg/mL X-gal and 0.5 mM IPTG. Positive colonies were screened by PCR with M13 universal primers using the described PCR profile [12]. Plasmid DNA from positive colonies was subsequently isolated by using a Plasmid DNA extraction kit (Biospin Bioer Technology Co. Ltd, China) and quantified on NanoDrop ND-1000 spectrophotometer (NanoDrop Technologies, USA). Plasmid DNA were confirmed by restriction enzyme digest mapping and the final products were shipped for Sanger sequencing (Macrogen Europe, UK).

### 2.10. Phylogenetic analysis

Sequenced amplicons were analysed, errors detected and corrected using DNAMAN software ver. 6 (Lynnon LLC., USA). Species were identified following with the Basic Local Alignment Search Tool (BLAST), which is implemented within the NCBI database (http://blast.ncbi.nlm.nih.gov/Blast.cgi.). When assigning an isolate, a species name, only BLAST search results showing >98% identity with a species' ITS sequence were considered.

Isolates were identified based on percentage identity to the *RefSeq* strain *A. flavus* GenBank: EU982012.1. Sequence comparisons of *RefSeq* strains and the isolates were aligned by using MUSCLE in MEGA ver. 6 [33]. The phylogenetic tree construction was inferred by using the Maximum Likelihood method under Tamura 3-parameter model [34]. The reconstructed phylogeny was tested for statistical support by bootstrapping using 1000 replicates [35]. Branches corresponding to partitions reproduced in less than 50% bootstrap replicates were collapsed. The percentage of replicate trees in which the associated taxa clustered together in the bootstrap test (1000 replicates) were shown above the branches [35]. Initial tree(s) for the heuristic search were obtained by applying the Neighbor-Joining method to a matrix of pairwise distances estimated using the Maximum Composite Likelihood (MCL) approach. A discrete Gamma distribution was used to model evolutionary rate differences among site [4 categories (+*G*, parameter = 1.6511)]. The analysis involved 60 nucleotide sequences where all positions containing gaps and missing data were removed prior to analysis. In the final dataset, there was a total of 174 positions. Evolutionary analyses were finally performed in MEGA ver.6 [33].

## 3. Results

### 3.1. Characterisation of the fungal isolates based on nit-mutants

**Figure 2.**
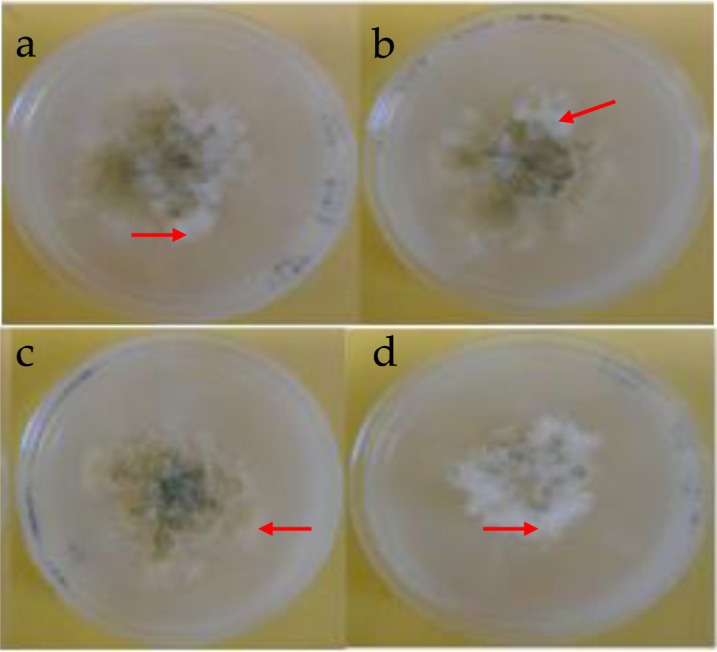
Plates showing VCGs tests. Four of the 37 *Aspergillus flavus* strains (a. HB-RNG 0024; b. KSM-MHN 0019; c. MC-UKA 0035; d. NC-MTT 0010) isolated from four regions of Kenya growing on minimal media (czapek dox agar) showing different growth patterns after 10 days of incubation at 30 °C. The extended radial hyphal tips growth (red arrows) is an indication of fast growing chlorate resistant sectors.

Three sets of biological replicates were performed. The *nit* mutants were selected as a tool for VCG testing. Accordingly, VCG tests revealed 4–8 chlorate resistant sectors growing radially to the edge of the plates. The sectors sporulated at different rates displaying characteristics of whitish mycelia growth at hyphal tips (see red arrows) of the colonies at the edges of the plates (Figure 2).

Different sources of nitrogen were tested on the *Nit* 1, *Nit* 2, *Nit* 3, *Crn* (chlorate resistant nitrate-utilizing) and wild type isolates (Table 1). The generated *nit* mutants were then classified based on three classes: *niaD* (nitrate non-utilising), *nirA* (nitrate and nitrite non-utilising) and *cnx* (nitrate hypoxanthine non-utilising). The results showed that the presence or absence of a nitrogen source plays a major role in determining the sensitivity tests of the *A. flavus* isolates. Total sporulation of *nit* mutant's isolates was observed mainly in NH_4_ source (data not shown) whereas the wild type exhibited growth in all except in ClO_3_ source. *Nit* 1, *Nit* 2, and *Nit* 3 displayed no growth in NO_3_ whereas wild type, *Nit* 1, *Nit* 2 and *Crn* exhibited growth on hypoxanthine.

**Table 1.** Sensitivity tests of Aspergillus flavus isolates and nit-mutants on different sources of nitrogen.

Isolate	Media				
	NH_4_	NO_3_	NO_2_	Hypoxanthine	ClO_3_
Wild type	+	+	+	+	-
*Nit*1	+	-	+	+	+
*Nit*2	+	-	-	+	+
*Nit*3	+	-	+	_	+
*Crn*	+	+	+	+	+

N/B: +. Presence of growth; -. Absence of growth.

### 3.2. Vegetative compatibility group testing of the Aspergillus flavus isolates

**Figure 3.**
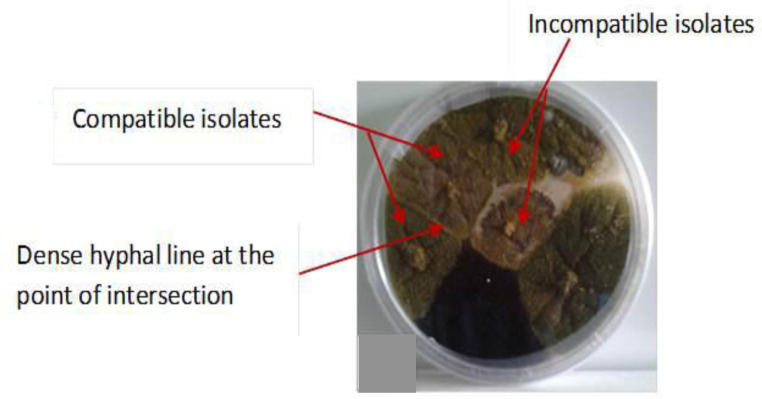
Agar plate showing heterokaryosis among *Nit* mutants of *A. flavus* isolates upon complementation tests, illustrating the formation of a dense line of hyphal/mycelia growth at the zone of intersection (Heterokaryon) after 36 days of incubation at 30 °C.

The findings showed isolates from each county were self-compatible within the county but incompatible between the counties with a few exceptions. Strong heterokaryon incompatibility was observed between Nandi county isolates (*n* = 6; 67%) and Makueni county (*n* = 3; 33%) isolates. Isolated strains from Nandi, Kisumu, and Homa Bay were vegetatively compatible, forming dense hyaline mycelia at the point of intersection (Figure 3).

Vegetative compatibility groups (VCGs) are typically identified by complementation of nitrate non-utilizing auxotrophs, and members of the same VCGs are considered to be members of the same clonal lineage. In this section, the main objective was to investigate the genetic diversity of the 37 *A. flavus* isolates through VCGs, phenotypes and to establish their distribution patterns across the study regions (Table 2).

**Table 2.** Some selected isolates illustrating the summary of phenotypic characteristics of *A. flavus* isolates, colony colour, colony diameter, phialides, exudates, sclerotia, conidia, VCGs, fluorescence, strain type and vesicle shapes.

County	Isolate	Colony colour	Phialides	Reverse colour	Ø (mm) CYA	Ø (mm) CZ	Exudates	Conidia shape	Vesicle shape	Sclerotia	UV (360nm)	VCGs	Strain type
Nandi	NC001-1	Green	none	Brown orange	25–25	15–20	Colourless	none	none	none	green		-
Nandi	NC002-1	Dull green	none	Dull yellow	55–60	20–25	Colourless	none	none	dark	blue green		S
Nandi	NC004-1	Light orange	uniseriate	Dull yellow	55–60	25–30	Colourless	rough surface	globose/spherical	none	blue green		-
Nandi	NC005-2	Dull green	none	Dull yellow	55–60	45–50	Brown yellow	none	none	none	green	4	-
Nandi	NC005-3	Dull yellow	uniseriate	Dull yellow	70–80	45–55	Green yellow	smooth surface	globose/spherical	dark	green		L
Nandi	NC006-1	Light brown	none	Dull yellow	55–60	35–40	none	none	none	none	green		-
Nandi	NC007	Dull green	biseriate	Dull yellow	60–70	45–50	Colourless	smooth surface	globose/spherical	none	blue		-
Nandi	NC009-2	Dull brown	biseriate	Dark tan	45–65	35–40	Brown	s rough surface	globose/spherical	dark	blue green		L
Nandi	NC0010-1	Dull brown	uniseriate	Dark tan	55–65	35–40	Brown	none	pyriform/globose	dark	blue		L
Kisumu	KSM0012	Dull green	uniseriate	Dull yellow	55–65	40–45	Pink brown	smooth surface	globose/spherical	none	green		-
Kisumu	KSM0013	Dull green	uniseriate	Dull yellow	55–60	45–50	Colourless	smooth surface	pyriform/globose	dark brown	green		L
Kisumu	KSM0014	Brown green	uniseriate	Brown yellow	55–60	45–50	Brown	smooth surface	globose/spherical	dark brown	blue		S
Kisumu	KSM0015	Dull yellow	uniseriate	Dull yellow	60–65	45–50	Green brown	smooth surface	spathulate/pyriform	dark brown	blue green		L
Kisumu	KSM0016-G	Dull green	uniseriate	Brown yellow	55–60	25–30	Colourless	smooth surface	spathulate/pyriform	none	blue	12	-
Kisumu	KSM0017	Brown green	uniseriate	Brown yellow	50–55	35–40	Pink brown	smooth surface	globose/spherical	dark brown	blue		L
Kisumu	KSM0017-Y	Brown/yellow green	uniseriate	Brown yellow	55–60	35–40	Pink brown	smooth surface	globose/spherical	dark brown	blue		S
Kisumu	KSM0019	Brown green	uniseriate	Brown yellow	55–60	35–40	Pink brown	fine rough	pyriform/globose	dark brown	blue green		L
Kisumu	KSM0020	Dull green	uniseriate	Brown yellow	45–50	35–45	Brown yellow	fine rough	pyriform/globose	dark brown	blue		S
Homa Bay	HB0021-1	Dull green	uniseriate	Dull yellow	55–65	40–45	Colourless	fine rough	globose/spherical	none	green		-
Homa Bay	HB0025	Dull green	uniseriate	Brown yellow	55–60	40–45	Brown yellow	fine rough	globose/spherical	dark	blue		L
Homa Bay	HB0026-Y	Brown-yellow	uniseriate	Dark tan	60–65	20–45	Brown yellow	fine rough	pyriform/globose	dark brown	blue green	8	S/L
Homa Bay	HB0027	Brown green	uniseriate	Brown yellow	60–70	35–40	pink brown	rough surface	globose/spherical	dark brown	blue green		S
Homa Bay	HB0028-1	Brown green	biseriate	Dull yellow	65–70	55–60	Brown yellow	fine rough	pyriform/globose	brown	green		L
Homa Bay	HB0028-2	Brown green	none	Brown yellow	65–70	55–60	Brown yellow	none	none	dark brown	blue green		L
Homa Bay	HB0029-1	Brown white	uniseriate	Dark tan	60–65	45–50	Brown yellow	smooth	spathulate/pyriform	dark	blue		S
Homa Bay	HB0030-1	Dull green	biseriate	Dull yellow	55–70	40–45	Colourless	smooth	pyriform/globose	none	blue		-
Makueni	MC0031	Dull green	biseriate	Dull yellow	55–60	45–50	Colourless	smooth	globose/spherical	none	blue		-
Makueni	MC0032-B	Brown green	uniseriate	Brown yellow	60–65	15–20	Brown	smooth	pyriform/globose	dark	blue green		L
Makueni	MC0033	Cotton white	biseriate	Dull yellow	55–60	35–45	Colourless	smooth	pyriform/spathulate	none	green		-
Makueni	MC0034-GY	Green-yellow	none	Dull yellow	55–60	40–50	Colourless	smooth	none	yellow brown	blue green	11	L
Makueni	MC0035-W	Cotton white	uniseriate	Dull yellow	55–60	45–50	Colourless	smooth	pyriform	none	blue green		-
Makueni	MC0035-G	Green	none	Yellow	60–70	30–35	Yellow green	none	none	none	blue		-
Makueni	MC0040	Dull green	uniseriate	Dull yellow	55–60	40–45	Colourless	smooth	pyriform	none	blue		-

N/B: CYA-Czapek Yeast Extract Agar; CZ-Czapek-Dox medium; UV-light at 360nm; s-small and L-large sclerotia

**Figure 4.**
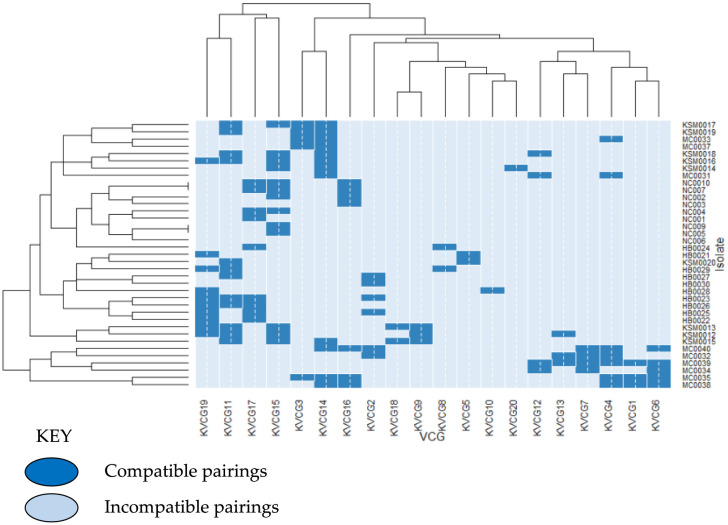
Heat map displaying the relationship between VCGs diversity and phenotype characters of *Aspergillus flavus* isolate from corn kernels from four different agroecological zones of Kenya. In brief, 37 isolates of *A. flavus* were used for VCG tester-pair development a total 740 tests conducted. The dark blue boxes represented compatible pairings whereas, light blue boxes show incompatibility pairings. R statistical software ver. 3.2.5 was used for data analysis.

We analyzed the data and generated the heat map. The results showed, the heat map generated identified clusters of the *A. flavus* isolates that were correlated with the isolate's phenotype (Figure 4). The light blue boxes represented incompatible pairings whereas, the dark blue boxes on the heat map (Figure 4) represented compatible pairings of a specific VCG for a strain. Inter-location VCGs genetic diversity across the surveyed regions were observed on the histograms. Certain isolates from Nandi and Homa Bay were observed to cluster together (compatible), whereas, others from Makueni and Kisumu were distributed within the two counties. This indicates the possibility of compatibility of the isolates between these agroecological zones. However, further research is needed to confirm the possible compatibility. The KVCG 10 (Homa Bay) and KVCG 20 (Kisumu) were restricted to specific regions (Figure 4). Most importantly, the heat map indeed reveals some clustering, but also reveals that very few isolates display the same compatibility/incompatibility pattern, indicating there is also diversity at the *het* (*vic*) loci within a geographic region.

### 3.3. Genomic DNA integrity and PCR amplification

Genomic DNA was extracted from the 37 isolates and the ITS regions were then amplified, cloned and sequenced. The PCR amplifications identified the genes of interest at 518 bp (Figures S1, S2). The ITS 1 and ITS 2 primers designed also amplified a 518 bp. Thus, the primers were specific and suitable for use either for *A. flavus* identification in the current study.

### 3.4. Phylogenetic analysis of *A. flavus* isolates

**Figure 5.**
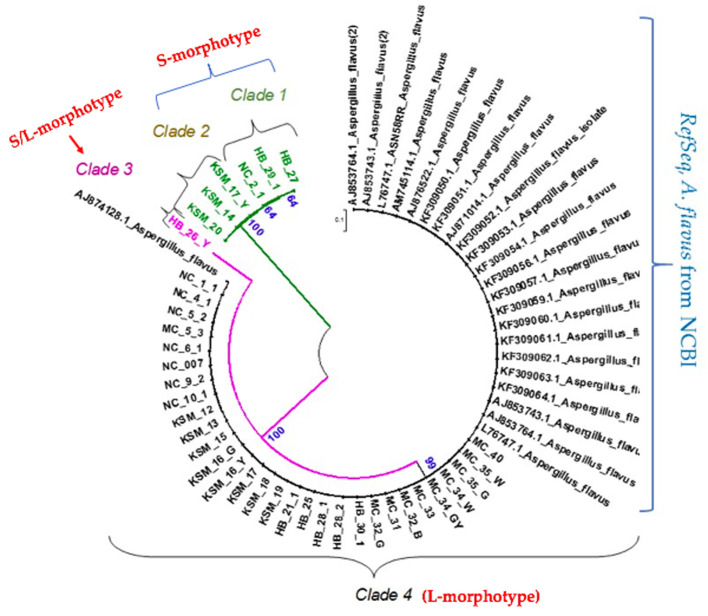
Maximum Likelihood Phylogenetic tree constructed from aligned DNA sequences of the ITS domain using MUSCLE, MEGA version 6. Nucleotide sequences generated are presented in bold, whereas the retrieved *RefSeq* from GenBank are labelled with their respective accession numbers as indicated in blue bracket. Clade 4 shows distribution patterns of the isolates with *RefSeq* from GenBank and surveyed counties (NC-Nandi county; KSM-Kisumu; HB-Homa Bay and MC-Makueni county and the numerical in front of the letters represent assigned codes). Some note for clarity on nomenclature, for example ‘MC-35-W and MC-35-G’ was an isolate from Makueni, which exhibited two colony colours; whitish (MC-35-W) and greenish (MC-35-G) colonies, ‘MC_34_GY’ refers to another isolate from Makueni with greenish-yellow colony, while ‘MC_32_B’ was a Makueni isolate with brownish colony.

Thirty-seven *A. flavus* isolates from Nandi, Kisumu, Homa Bay and Makueni were examined previously (section 3.2) to clarify their taxonomic status based on their VCGs and genotypes. BLASTn hit and a phylogenetic analysis of ITS1 and ITS2 sequences of the isolates were evaluated. BLASTn hit results showed identity similarity level ranging from 99% to 100% identity to *A. flavus RefSeq* sequences from NCBI, thus confirming their identity as *A. flavus* isolates. ITS primers discriminated *A. flavus* isolates; 10.8% (*n* = 4) as 100% bootstrap support, 81.1% (*n* = 30) as 99% and 8.1% (*n* = 3) as 64% bootstrap support, respectively (Figure 5). Certain isolates (HB026Y, clade 3) was discriminated as 100% bootstrap support from the rest of the isolates (Homa Bay, Nandi, Kisumu and Makueni regions) and formed its own clade from the members of the group as revealed by Maximum Likelihood (ML) and Tamura 3-parameter model. The phylogeny reconstruction tree revealed isolates (KSM014, KSM020, KSM017Y and HB026Y) formed highly supported clusters with 100% bootstrap support (Clade 1 and clade 3) (Figure 5). In the Maximum Parsimony model method, the four isolates also exhibited similar trend with 100% bootstrap support as in ML model.

Further phylogenetic assessment showed the *RefSeq* from the GenBank clustered at 99% bootstrap support with isolates across the regions; Makueni (100%, n = 10), Homa Bay (65%, n = 5), Kisumu (72%, n = 8), and Nandi (91%, n = 7) (Figure 5; clade 4). Clade one isolates were from Homa Bay (n = 2) and Nandi (n = 1) region exhibiting 64% bootstrap support. Clade 2 isolates were observed to have originated from Kisumu exhibiting bootstrap support 100% (Figure 5). Interestingly, none of the isolates from Makueni region clustered in clade 1, 2 and 3. The findings were similar to Samson *et al.* [35], who found that, *Aspergillus* is a diverse genus and the species occurs worldwide in various habitats*. A.* *flavus* isolates from Kisumu region were observed to be more closely related to strains from Nandi than to those from Homa Bay region. Clade 1 and 2 isolates originated from regions with little or no risk of aflatoxicosis over the years.

It is important to note that we did not see significant cross talk between the data obtained from the heat map, VCGs, phenotypes and phylogenetic analysis showing associations amongst the isolates from different regions (Figures 4, 5). However, it is interesting to see the geographical dependence of the VCG variations, which will add knowledge for a better understanding of the taxonomic and phenotypic variation among the *A. flavus* isolates.

## 4. Discussion

The ability to produce aflatoxin is not a useful character in discriminating species within *Aspergillus* section *Flavi*. Some authors [36] observed wide variation in *A. flavus* populations and [5] (5)found that many strains can lose their ability to produce aflatoxin overtime.

Some chlorate-resistant sectors were observed to grow very rapidly and radially from the colony while others grew more slowly (Figure 2). These different growth patterns could be attributed to chlorate, which is an analogue of nitrate and preferred by fungi for growth [4],[29],[37],[38]. Upon reduction by the nitrate reductase, the chlorate would become chlorite which is poisonous substance to fungi [4],[29],[37],[38] hence the different growth patterns.

Certain isolates from Nandi and Homa Bay were observed to cluster together (compatible), whereas, others from Makueni and Kisumu were distributed within the two counties (Figures 4, 5). This indicates the possibility of likelihood or compatibility of the isolates between these agroecological zones. KVCG 10 (Homa Bay) and KVCG 20 (Kisumu) were restricted to specific regions (Figure 4). This has been remarkably observed on the heat map (Figure 4) generated showing the relationship between the VCGs, phenotypic characters and clustering in the phylogenetic tree constructed (Figure 5). The results showed that, the heat map generated identified clusters of the *A. flavus* isolates that were correlated with the isolate's phenotype (Figure 4). The light blue boxes represented incompatible pairings whereas, the dark blue boxes on the heat map (Figure 4) represented compatible pairings of a specific VCG for a strain. The higher the intensity of the blue colours on the heat map, the more compatibility of the isolates with regard to the VCGs characteristics and genetic diversity. Inter-location VCGs genetic diversity across the surveyed regions were observed on the histograms.

Interestingly, isolate NC006 was found to be incompatible with every single tester strain we used. We believe the isolate could then represent a new VCG on its own. Hence, we strongly believe that the isolate is novel and could be registered as a new tester strain.

Our findings were similar to studies conducted by Horn & Greene, [39] and Pildain *et al.* [4] who noted that *A. flavus* strains are composed of many VCGs. Additionally, VCG diversity could be interrelated to variations in specialized regions within the *A. flavus* populations displaying competitive advantages in specific agro-ecological zones. Pildain *et al.* [4] and Atehnkeng *et al.* [16]; Atehnkeng *et al.* [11] further demonstrated that, frequencies of strains and VCGs vary by farm land and crop, and that some VCGs are frequently isolated whereas others are uncommon. VCG distribution frequency and diversity indices of the isolates were closely linked to specific regions [13]. Bock *et al.* [41] and Cotty, [42] suggested that different *A. flavus* lineages might be distributed according to niche speciality adaptations and have competitive advantages in specific regions, soils, hosts or seasons.

Phylogenetic analysis of the ITS1 and ITS2 domain sequences exhibited variable clustering with some isolates clustered together whereas others clustered with the *RefSeq* from the GenBank (Figure 5). Clusters had bootstrap support ranging from 64%–100% (Figure 5). Majorly, isolates from Makueni region clustered on clade 4 apart from clades 1, 2 and 3 with most isolates from the three other regions at 99% bootstrap support to the *RefSeq* from NCBI. Moreover, they were closer to NCBI sequences thus, a clear indication of close relatedness and homogeneity. Clades 1, 2 and 3 were observed to be isolates with higher possibility of production of aflatoxins, with clade 3 suspected to belong to S/L-morphotype whereas clades 1 and 2 belong to S-morphotype (Figure 5). The current findings were similar to the analysis of the ITS1 and ITS2 sequences conducted by Gonçalves *et al.* [44] which revealed high molecular heterogeneity of *A. flavus* strains and their close relatives. Studies conducted previously [12], demonstrated isolate HB026Y to have characteristics of a S/L morphotype and this could have been the reason for discrimination to isolated clade (Figure 5, clade 3) and similar trend was observed on the heat map (Figure 4). *A. flavus* is a genetically complex species [4],[45],[46] and numerous cryptic species have been identified [44]. A phenotypic and molecular investigations conducted by Gonçalves *et al.* [44] on a set of isolates of *A. flavus* revealed that primer sets used gave respective varying amplicon lengths. The ITS primers gave an amplicon that was 520–535 bp in length, *amd* was 540–550 bp, *omtA* was 465–480 bp and BT2 was 508–522 bp and this was similar to our findings which gave similar amplicon size polymorphism for *A. flavus* strains from the four different climatic regions of Kenya.

## 5. Conclusion

Strains from Nandi, Kisumu, and Homa Bay were vegetatively compatible. Makueni's *A. flavus* isolates were more diverse compared to isolates from the three regions. Kisumu isolates were closely related to strains from Nandi and Homa Bay. Within *A. flavus*, the ITS1 and ITS2 markers did not reveal significant information on intra-species differentiation. The high genetic similarity of species of *Aspergillus* together with the high degree of intra-specific inconsistency might have led to the inability to detect the isolates, and in the future other molecular markers for distinguishing the strains at species level should be considered.
