# Supplementary material for: Molecular and Vegetative Compatibility Groups Characterization of Aspergillus flavus Isolates from Kenya
Source: AIMS Microbiol. 2020 Jul 31;6(3):231–49. doi: 10.3934/microbiol.2020015 (PMC7595839; doi:10.3934/microbiol.2020015)
Supplement: Supplementary file 1 [file microbiol-06-03-015-s001.pdf]

AIMS Microbiology, 6(3): 231–250.
DOI: 10.3934/microbiol.2020015
Received: 21 April 2020
Accepted: 06 July 2020
Published: 30 July 2020

http://www.aimspress.com/journal/microbiology

Research article

Molecular and Vegetative Compatibility Groups Characterization of *Aspergillus flavus* Isolates from Kenya

Alfred Mitema[1,2] and Naser Aliye Feto[1,]*

[1] OMICS Research Group, Department of Biotechnology, Vaal University of Technology, Vanderbijlpark 1911, South Africa
[2] School of Biological Sciences, University of Nairobi, Nairobi, Kenya

***Correspondence:** anaser22@yahoo.com; naserf@vut.ac.za.

Supplementary materials

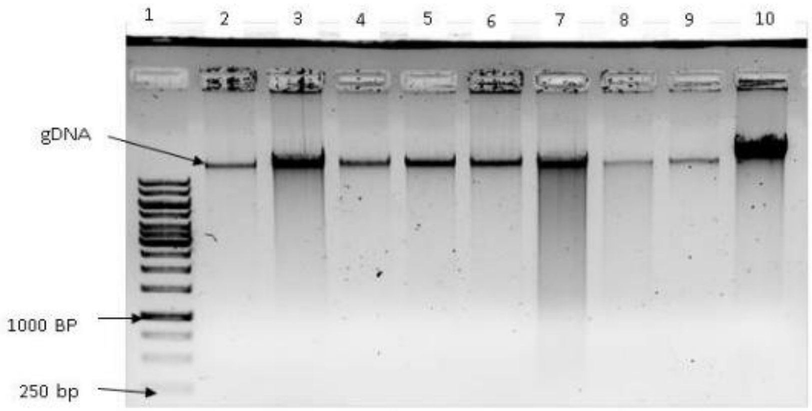

Figure S1. Gel electrophoresis of gDNA extracted from *Aspergillus flavus* strains isolated from maize kernels collected from four different climatic regions of Kenya assessed on 1% agarose/EtBr gel run at 80 volts for 45 min. Lanes: 1. 1 Kb Ladder; 2. NC01; 3. NC04; 4. KSM012; 5. KSM016 Y; 6. HB025; 7. HB027; 8. MC031; 9. MC034W; 10. MC040 G. (NC: Nandi county; KSM: Kisumu; HB: Homa Bay; MC: Makueni).

PCR amplifications identified the genes of interest at 518 bp (blue arrow) (Figure 4). The ITS 1 and ITS 2 primers designed also amplified a 518 bp. Thus, the primers were specific and suitable for use either for *A. flavus* identification in the current study.

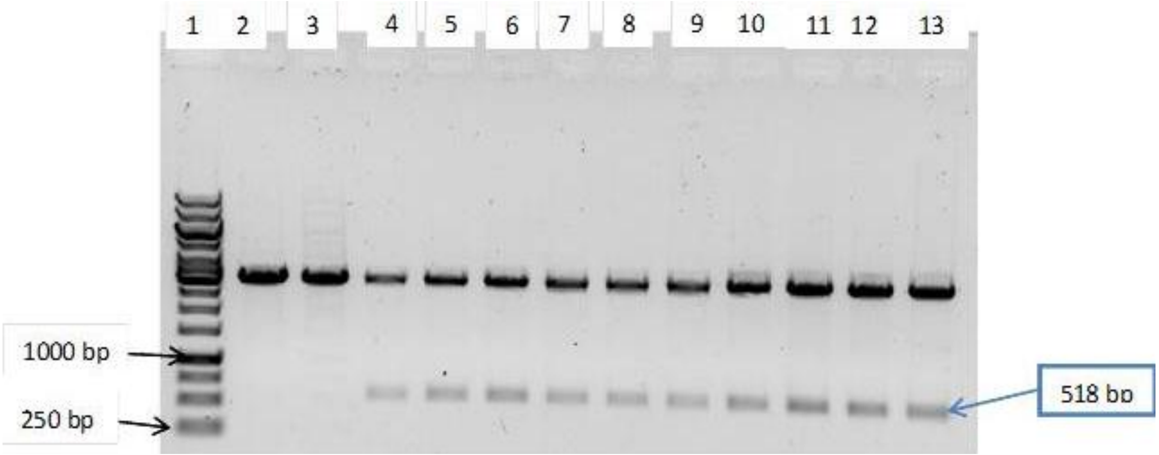

Figure S2. Images of restriction digests with *Not1* enzyme amplified on PCR machine using primers (M13F and ITS1/ITS2R) identified the genes of interest at 518 bp run on 1 % agarose/EtBr gel at 80 volts for 45 minutes. 1. 1 Kb Ladder; 2. NC01 (-); 3. KSM012 (-); 4. NC01; 5. NC04; 6. KSM012; 7. KSM016; 8. HB025; 9. HB027; 10. MC031; 11. MC034; 12. MC035; 13. MC040. Lanes 1. 1kb marker; 2 and 3 shows undigested isolates whereas, lanes 4–13, positive digests. (NC: Nandi county; KSM: Kisumu; HB: Homa Bay; MC: Makueni).

AIMS Press

© 2020 the Author(s), licensee AIMS Press. This is an open access article distributed under the terms of the Creative Commons Attribution License (http://creativecommons.org/licenses/by/4.0)
